# MOTIV Bioresorbable Scaffold in Below-The-Knee Artery Disease: European Post-Market Pilot BTK Trial: 36-Month Results

**DOI:** 10.1007/s00270-025-04202-8

**Published:** 2025-09-22

**Authors:** Michel J. Bosiers, Thomas Rand, Raman Uberoi, Henrik Schroeder, Nasser Malyar, Dierk Scheinert, Andrej Schmidt

**Affiliations:** 1 Department of Vascular Surgery, St. Franziskus-Hospital, Münster, Germany; 2 Department of Vascular Surgery, University Hospital Bern, University of Bern, Freiburgstrasse 18, 3010 Bern, Switzerland; 3 Institute for Interventional and Diagnostic Radiology, Klinik Floridsdorf, Vienna, Austria; 4 Karl Landsteiner Institute for Interventional and Diagnostic Radiology, Vienna, Austria; 5 Department of Radiology, John Radcliffe Hospital, Oxford University Hospitals NHS Trust, Oxford, UK; 6 Institute for Interventional and Diagnostik Radiology at the Jewish Hospital Berlin, Berlin, Germany; 7 Department of Cardiology, University Hospital Muenster, Muenster, Germany; 8 Department of Angiology, University Hospital Leipzig, Leipzig, Germany

**Keywords:** CLTI, Bioresorbable scaffolds, Drug-eluting technology, BTK

## Abstract

**Purpose:**

THE primary objective of the MOTIV BTK PILOT STUDY WAs to evaluate the immediate and long-term safety and efficacy of the MOTIV® sirolimus-eluting bioresorbable scaffold (Reva Medical, San Diego, California, USA) in below-the-knee (BTK) arteries for the treatment of patients with rest pain or minor tissue loss (critical limb-threatening ischemia (CLTI)).

**Materials and Methods:**

This is a prospective, single-arm, multi-center trial of a novel drug-eluting bioresorbable scaffold with a new scaffold material (Tyrocore®), which includes an iodinated, polycarbonate copolymer of tyrosine analogs and has a surface coating of the same Tyrocore material and the antiproliferative drug sirolimus. Fifty-eight patients were included between August 2019 and July 2021. The primary efficacy outcome measure was primary patency at 12 months. The primary safety outcome measure was freedom from serious device-related adverse events at 30 days. Secondary outcome measures were immediate technical success, primary patency at 24 and 36 months, clinically driven target lesion revascularization rate (CD-TLR) and limb salvage at 12, 24, and 36 months. Follow-up was performed at 1, 6, 24, and 36 months, including clinical assessment and core laboratory adjudicated color duplex ultrasound.

**Results:**

Seventy-six MOTIV scaffolds were implanted in 60 study limbs with an average lesion length of 29.5 mm. Primary patency at 12, 24, and 36 months was 88.3%, 81.7%, and 80% (with numbers of limbs at risk being 43, 38 & 30) respectively. The 30-day adverse event rate was 1.7%. Technical success was achieved in 99%. At 3 years, freedom from CD-TLR was 93% and limb salvage rate was 95%.

**Conclusion:**

The 36-month results of this pilot MOTIV BTK study demonstrated favorable safety and effectiveness performance in CLTI patients with BTK atherosclerotic disease.

Level of Evidence: Level 3: non-randomized controlled cohort study.

**Graphical Abstract:**

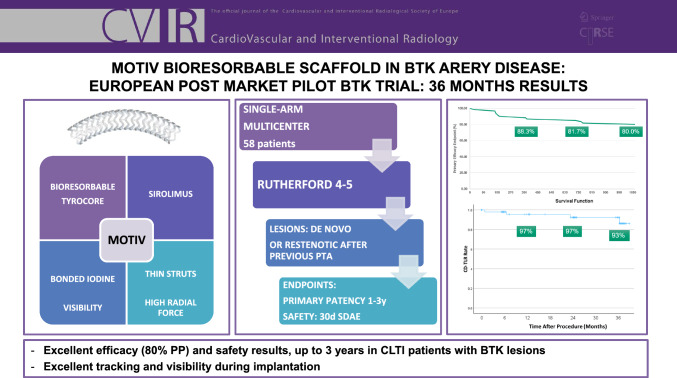

## Introduction

The prevalence of peripheral arterial disease (PAD) is rising, with over 230 million people affected worldwide [1]. In patients with chronic limb-threatening ischemia (CLTI), prompt revascularization is essential to prevent limb loss. Endovascular procedures are commonly used to revascularize below-the-knee (BTK) arteries in CLTI patients.

Early techniques, such as plain balloon angioplasty (POBA) and bare-metal stenting (BMS), initially showed high success rates but were significantly limited by high restenosis rates [2, 3]. Additionally, angioplasty is hindered by issues like elastic recoil and an increased risk of arterial dissection [4]. To overcome these limitations, newer technologies have been introduced, including drug-coated balloons (DCBs) and drug-eluting stents (DES). While DCBs have demonstrated improved patency rates in femoropopliteal arteries, their effectiveness in BTK vessels has been inconsistent [5–7]. For example, in the Lutonix-BTK [5] and BioLUX P-II [7] trials, no difference in freedom from amputation at 12 months was observed between the DCB and POBA groups (86.7% vs. 84.7% and 60.4% vs. 60.9%, respectively). Across multiple randomized controlled trials, DES demonstrated superiority over angioplasty or BMS, particularly in treating short, isolated lesions [8–11]. In the ACHILLES [8] trial, the primary patency rate at 12 months was 75% for DES vs. 57.1% for POBA. Similarly, the YUKON [9] and DESTINY [10] trials reported rates of 80.6% vs. 55.6% and 85% vs. 54%, respectively, comparing DES to BMS.

However, the presence of a stent can complicate future reinterventions.

Infrapopliteal artery disease presents unique challenges due to long occlusions and severe calcification, which hinder drug absorption into the arterial wall [12, 13]. Additionally, the small diameter and lower flow rates of these vessels, compared to coronary arteries, may limit DES efficacy.

To address these challenges, innovative vessel preparation techniques have been developed to optimally prepare the arterial wall before stent or balloon deployment in an effort to improve results. Another advancement is the development of drug-eluting resorbable scaffolds, which provide structural support to address mechanical failures while serving as a platform for antiproliferative drug delivery during the restenotic phase. Their gradual resorption supports vessel remodeling and may reduce complications associated with permanent implants.

The MOTIV scaffold is a novel device made from Tyrocore® (Reva Medical, San Diego, California, USA), a bioresorbable material that combines scaffold support with sirolimus drug delivery to minimize vessel irritation and intimal hyperplasia.

This pilot study aims to evaluate the short- and long-term outcomes of this new drug-eluting bioresorbable scaffold in treating CLTI patients with BTK disease.

## Materials and Methods

The MOTIV BTK pilot trial is a prospective, single-arm, multi-center, post-market study, designed to evaluate the safety and effectiveness of the MOTIV Peripheral Vascular Bioresorbable scaffold. The MOTIV scaffold is a combination device composed of Tyrocore, a bioresorbable material, coated with the antiproliferative drug Sirolimus at a concentration of 1.97 µg/mm^2^. Tyrocore is composed of tyrosine analogs (desaminotyrosine) and biocompatible hydroxy esters. It features an iodinated diphenol and a low-molecular-weight polylactic acid diol oligomer in an 8:1 ratio. The phenyl ring of the iodinated diphenol provides a robust molecular framework, contributing to Tyrocore’s exceptional tensile strength. Its ability to maintain flexibility while preserving strength stems from its distinctive composition and high molecular weight. The covalently bonded iodine enhances radiopacity, ensuring visibility under fluoroscopy. The strut thicknesses are 95 μm, 105 μm, and 115 μm for diameters of 2.5 mm, 3.0 mm, and 3.5 mm, respectively, and all devices are available in lengths up to 60 mm. The diameters from 2.5 to 3.0 mm may be safely post-dilated 0.75 mm beyond their nominal diameter, while 3.5 mm scaffolds can be expanded to 0.5 mm beyond their nominal diameter. After implantation, the MOTIV scaffold provides mechanical support for six months, as demonstrated by REVA Medical in a series of preclinical evaluations, and then gradually resorbs over four years. It is 6 French compatible and mounted on a rapid exchange balloon.
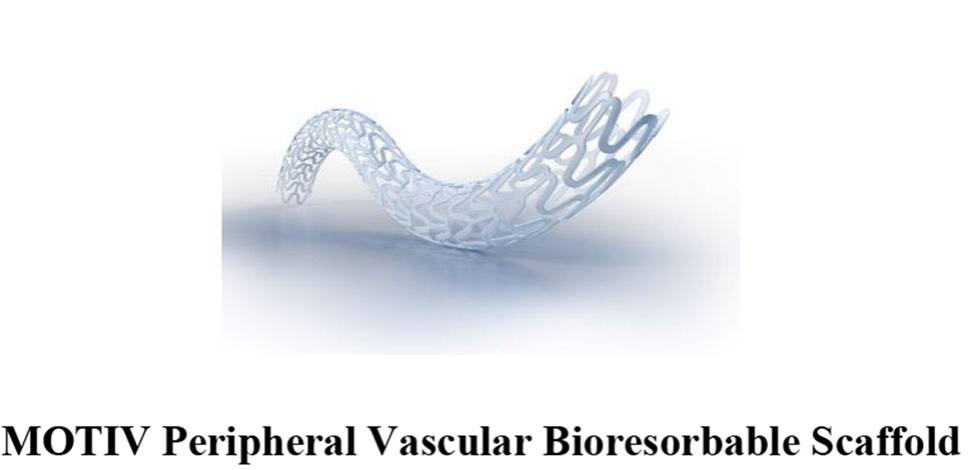


The MOTIV pilot study is registered at ClinicalTrials.gov under NCT03987061.

The trial planned to enroll 58 patients across nine clinical investigation centers. The name of the PI of each participating center can be found in Table 1. A sample size of 50 evaluable patients was chosen to provide an adequate estimate of “true ” rate of treatment success at 12 months, with an additional eight patients being added to offset for expected loss to follow-up and early study termination.

**Table 1:** PI and participating center

Site PI	Site
Bosiers Michel (lead PI)	St. Franziskus Hospital Muenster, Germany
Willecke Florian	Herz- und Diabeteszentrum, Bad Oeynhausen, Germany
Reimer Peter	Klinikum Karlsruhe, Germany
Balzer Jörn	Katholische Klinikum Mainz, Germany
Rand Thomas	Klinik Floridsdorf, Austria
Lichtenberg Michael	Klinikum Hochsauerland Arnsberg, Germany
Scheinert Dierk	University Hospital Leipzig, Germany
Maylar Nasser	University Hospital Muenster, Germany
Schroeder Henrik	Jewish Hospital Berlin, Germany

Inclusion and exclusion criteria can be found in Table 2. In summary, patients with Rutherford 4 and 5 and BTK lesions could be enrolled. Two sorts of lesions were included in the study: de novo lesions requiring a scaffold (dissection or recoil) or restenotic lesions after previous PTA, suitable for endovascular therapy. Target vessel diameter estimated visually had to be ≥ 2.5 mm and ≤ 3.5 mm. The total lesion length that required scaffolding had to be < 100 mm. Longer lesions could be treated with angioplasty, as long as the total amount of scaffolding did not exceed 100 mm. In cases where total lesions coverage required two or more scaffolds, the MOTIV devices were placed in an edge-to-edge fashion, without overlap, under direct angiographic visualization. Inflow lesions could be managed at the operator’s discretion.

**Table 2:** In- and exclusion criteria

General inclusion criteria	Exclusion criteria
Patient is willing to comply with specified follow-up evaluations at the specified times	The reference segment diameter is not suitable for the available scaffold design
Patient presenting with rest pain or minor tissue loss (Rutherford classification from 4 to 5)	Untreated flow-limiting aortoiliac stenotic disease
Patient is > 18 years old	Perioperative unsuccessful ipsilateral percutaneous vascular procedure to treat inflow disease just prior to enrollment
Patient understands the nature of the procedure and provides written informed consent, prior to enrollment in the study	Any previous surgery in the target vessel
Patient has a projected life expectancy of at least 24 months	Aneurysm located at the target vessel
Patient is eligible for treatment with the MOTIV™ Bioresorbable Scaffold	Non-atherosclerothic disease resulting in occlusion (e.g., embolism, Buerger’s disease, vasculitis)
Male, infertile female, or female of child bearing potential practicing an acceptable method of birth control with a negative pregnancy test within 7 days prior to study procedure	Severe medical comorbidities (untreated CAD/CHF, severe COPD, metastatic malignancy, dementia, etc.) or other medical condition that would preclude compliance with the study protocol or 2-year life expectancy
*Angiographic inclusion criteria*	Major distal amputation (above the transmetatarsal) in the study or non-study limb
De novo lesion or restenotic lesion after PTA in the infrapopliteal arteries, suitable for endovascular therapy	Septicemia or bacteremia
Target vessel diameter visually estimated to be ≥ 2.5 mm and ≤ 3.50 mm	Any previously known coagulation disorder, including hypercoagulability
Guidewire and delivery system successfully traversed the lesion	Contraindication to anticoagulation or antiplatelet therapy
Total target lesion is maximally 100 mm	Known allergies to scaffold or scaffold component
Definition of Target Lesion is: a) de novo or Restenotic lesion after PTA or b) a residual flow-limiting dissection or restenosis after PTA of a longer lesion	Known allergies to contrast media that cannot be adequately premedicated prior to the study procedure
	Patient with known hypersensitivity to heparin-induced thrombocytopenia (HIT) type II
	Currently participating in another clinical research trial

### Procedure

Vascular access was obtained per the investigators’ standard clinical practice. Lesion crossing with the guidewire into the distal vessel was required prior to lesion preparation. All patients received 5,000 units of heparin at the start of the procedure.

Pre-dilatation was performed with a non- or semi-compliant balloon with a minimum reference to the artery ratio of 1:1 and with a maximum diameter of 3.5 mm under angiographic guidance.

Post-scaffold balloon dilatation was mandatory in all cases using a non- or semi-compliant balloon matching the reference diameter at ≥ 12 atm.

Operators were not blinded to treatment in follow-up assessments. All patients underwent clinical and duplex ultrasound follow-up at 1, 6, 24, and 36 months, with core lab adjudication. Data collected included patient demographics, medications, physical examination findings, ankle-brachial index (ABI), Rutherford score, and color flow Doppler ultrasound (pre-, intra-, and post-procedure measurements). Angiography, CT angiography (CT Angio), and MR angiography (MR Angio) were performed at the investigator’s discretion. Concomitant medication during hospital stays and follow-up was recommended as Clopidogrel 75 mg/daily for at least 3 to 4 months and lifelong aspirin: 75 to 300 mg daily.

Primary and secondary outcome measures were defined as:

*Primary* outcome measures:

1. Efficacy outcome measure: Primary patency rate (PPR) at 12 months, with PPR defined as no evidence of at least 50% restenosis or reocclusion within the originally treated lesion based on color flow duplex ultrasound (CFDU) measuring a peak systolic flow velocity ratio < 2.5 as determined by an independent Core Lab.
2. Safety outcome measure was defined as serious device-related adverse events within 30 days post-procedure.

*Secondary* outcome measures:

1. Technical success, defined as successful MOTIV implantation with a post-procedure residual angiographic stenosis < 30%
2. PPR rate a 1-, 6-, 24-, and 36-month follow-up as defined in the efficacy outcome measure above.
3. Clinical driven target lesion revascularization (CD-TLR) as a repeat intervention to maintain or re-establish patency within the region of the treated arterial vessel plus 5 mm proximal and/or distal to the treated lesion
4. Limb salvage rate, defined as the absence of major amputation.
5. Clinical success at follow-up was defined as an improvement of Rutherford classification at follow-up of at least one class compared to the pre-procedure Rutherford classification.

## Results

A total of 58 patients were enrolled between August 2019 and July 2021. Seventy-six MOTIV scaffolds were implanted in 60 study limbs. A significant portion of the patients were men (84%) with a mean age of 77 years (range 54–95). The majority of patients were Rutherford stage 5 (75%). Other comorbidities can be found in Table 3. The average lesion length was 29.5 mm (range 5-100 mm) with a diameter of 3 mm (range 2.5–3.5 mm). The total average scaffolding length was 26.92 mm (range 18-48 mm) with an average diameter of 3.1 mm (range 2.5–3.5 mm). The majority of limbs received the MOTIV scaffold for short de novo or restenotic lesion after angioplasty (61.7%). Other lesion characteristics can be found in Table 4.

**Table 3:** Patient demographics

Demographics	Mean or % (n/N)		
Male (96)	84% (49/58)		
Age (min–max)	77 (54–95) years		
Comorbid Conditions	% (n/N)		
Smoking		*Endocrine*	
Past	24.1% (14/58)	Diabetes type 1	17.2% (10/58)
Current	19% (11/58)	Diabetes type 2	42.3% (28/58)
Vascular		*Renal*	
Hypertension	81% (47/58)	Renal Insufficeny	43.1% (25/58)
Medicated	70.7% (41/58)	Dialysis	3.5% (2/58)
Previous peripheral Vascular interventions	51.7% (30/58)	*Obesity*	
Previous coronary Interventions	41.4% (24/58)	BMI (avg. min–max)	27 (17.93–38,09)
Previous cerebrovascular Interventions	3.5% (2/58)	Hypercholesterolemia	46.6% (27/58

**Table 4:** Lesion Characteristics

	Average or %, n/N (min – max)
Lesion length	29.5 mm (5.00–100.00)
Ref vessel diameter	3.0 mm (2.50–3.50)
Total scaffolds implanted	76
More than 1 MOTIV stent implanted	21.7% (13/60)
Pre-dilatation (obligatory)	100% (60/60)
Post-dilatation (obligatory)	100% (60/60)
	*Average or %, n/N (min – max)*
*Target lesion definition*	
De novo lesion or restenotic lesion after PTA	61.7% (37/60)
Residual flow-limiting dissection or restenosis after PTA of a longer lesion	38.3% (23/60)
*Target lesion pre-conditions*	
Ulceration	11. 7% (7/60)
Calcified lesion	46.7% (28/60)
Thrombus	30.0% (18/60)
Dissection	21.7% (13/60)

The average procedure time was 76.41 min (range 29-180 min) and the MOTIV scaffold was implanted in a variety of BTK arteries (Table 5).

**Table 5:** Procedural Characteristics

	Average or %, n/N (min – max)
Procedure time	76.4 min (29–180)
Fluoroscopy time	17.2 min (4.0–56.0)
Amount of contrast	86.7 mL (20–350)
Study limb (left:right)	55%: 45%
Rutherford 4:5:	25%: 75%
Artery treated:	
Anterior tibial artery (ATA)	36.7% (22/60)
Posterior tibial artery (PTA)	11.7% (7/60)
Peroneal artery (PA)	21.7% (13/60)
Tibioperoneal tract (TT)	20.0% (12/60)
ATA + TT	1.7% (1/60)
PTA + TT	1.7% (1/60)
PA + TT	6.7% (4/60)
Inflow lesion treated (%)	26.7% (16/60)
Additional lesions treated (%)^1^	43.3% (26/60)

^**1**^Additional lesions are lesions treated within the same limb in addition to the study lesion

A total of 43, 38, and 30 limbs completed follow-up at 12, 24, and 36 months, respectively, and 43, 35, and 24, respectively, received duplex ultrasound.

**Fig. 1:**
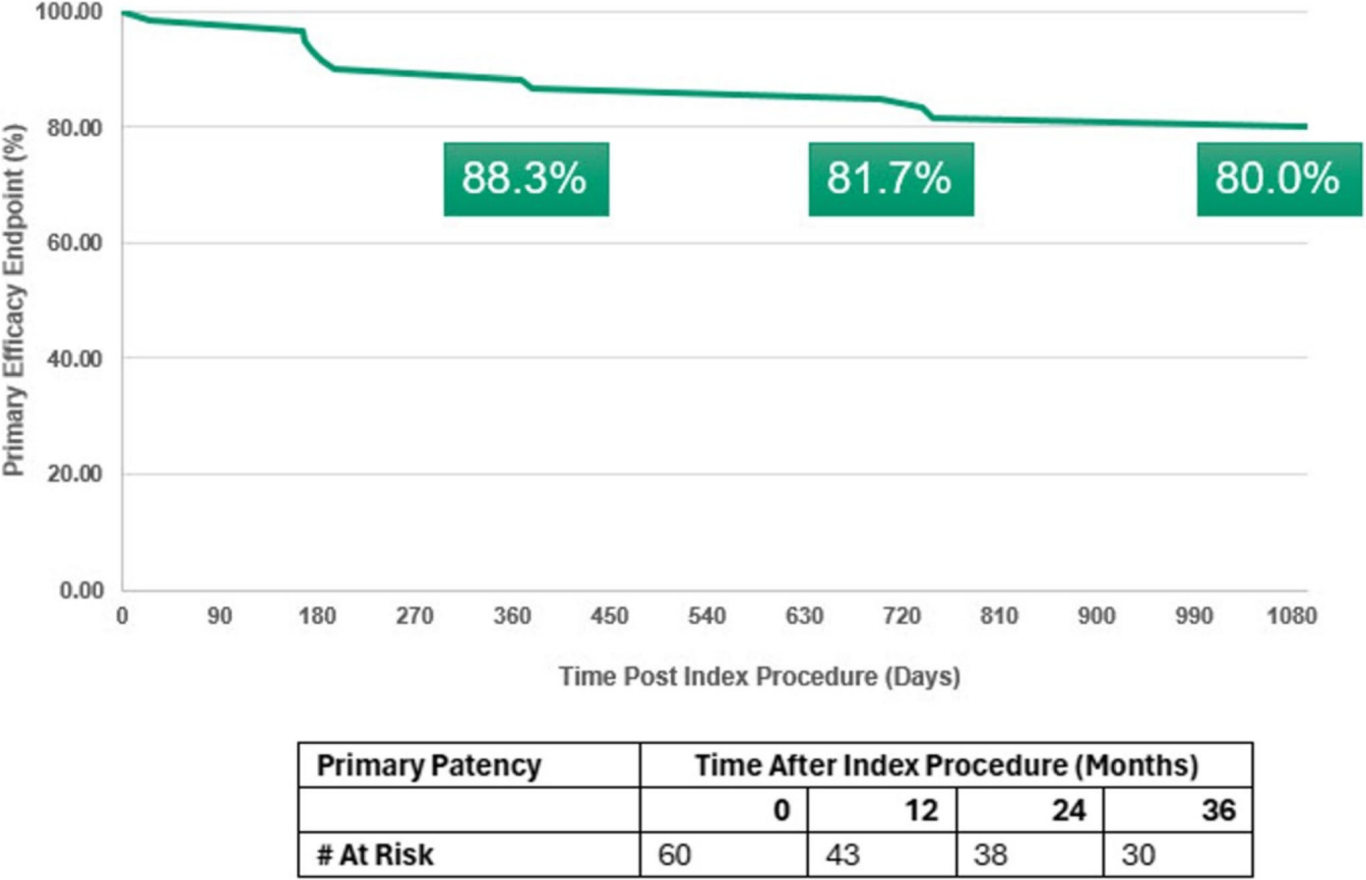
Kaplan–Meier curve for primary patency rate throughout 36 months

Primary Patency at 12, 24, and 36 months was 88.3%, 81.7%, and 80% respectively (Fig. 1).

**Fig. 2:**
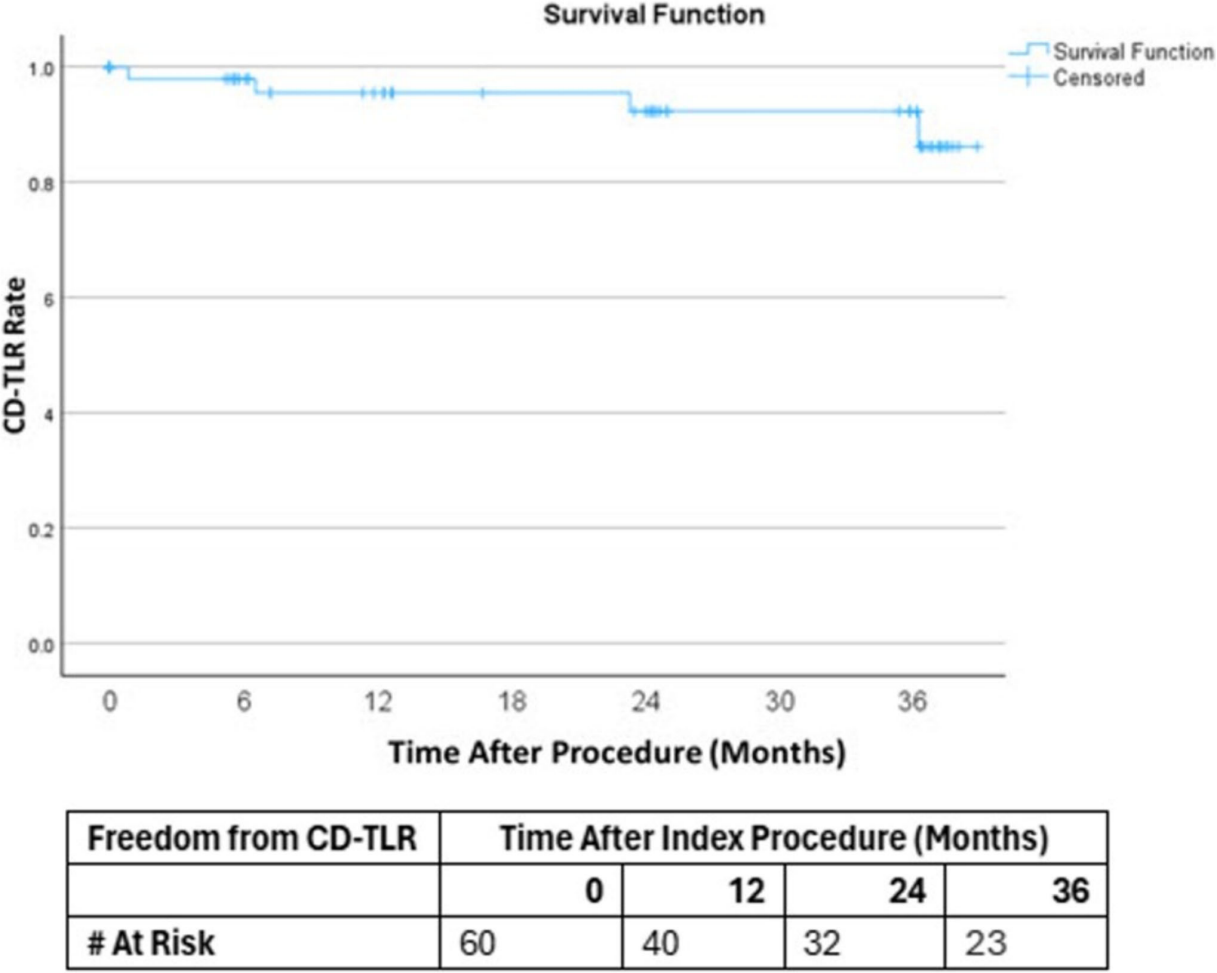
Kaplan–Meier curve for freedom from CD-TLR

The 30-day adverse event rate was 1.7%. Technical success was achieved in 99%. Freedom from CD-TLR was 97%, 97%, and 93% at 1, 2, and 3 years, respectively (Fig. 2). One patient had an occlusion at day 25, which was treated with PTA and a stent. One patient had a stenosis at day 196 and was treated with PTA and an additional MOTIV scaffold. One patient was treated with DCB and a stent at 23 months. Lastly, one patient required DCB treatment because of a restenosis at 36 months post baseline procedure.

The limb salvage rate was 95% at 3 years.

There were 3 amputations, all of them Rutherford 5 at baseline. One patient had a lower leg amputation at 1.5months due to poor wound healing, unrelated to the MOTIV scaffold as the one month PSVR was 1.7 and the visual estimate of stenosis was < 30%. The second patient had an amputation of study limb at 6 months due to a septic wound infection, reported as unrelated to the MOTIV scaffold. The third patient had an amputation of the study limb at 20 months due to a reocclusion of a non-target vessel after bypass and thrombectomy failure, reported as unrelated to the MOTIV scaffold.

During the study period, 16 patients died (28%). None of the deaths were study device or study intervention related.

**Fig. 3:**
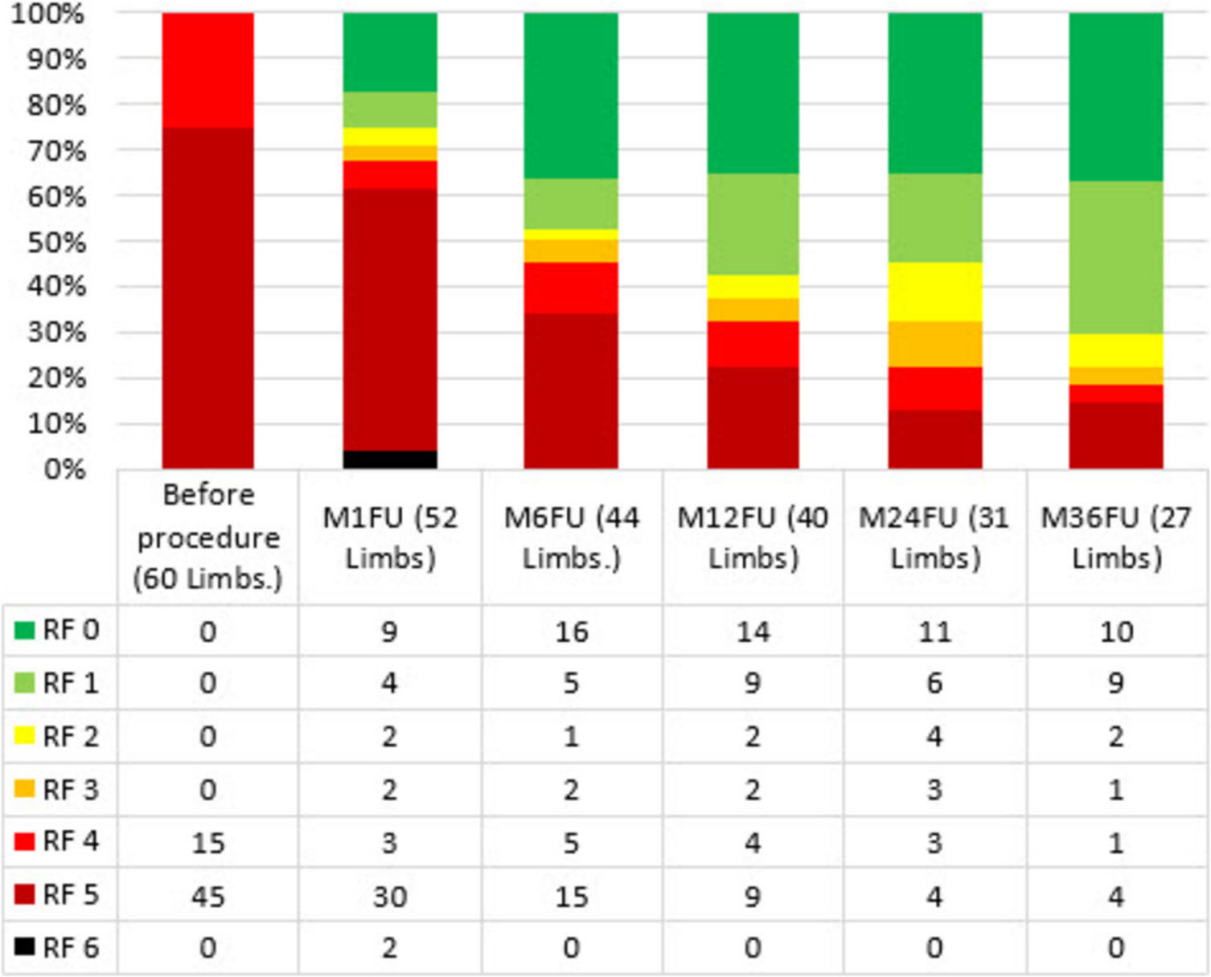
Rutherford Evolution

**Fig. 4:**
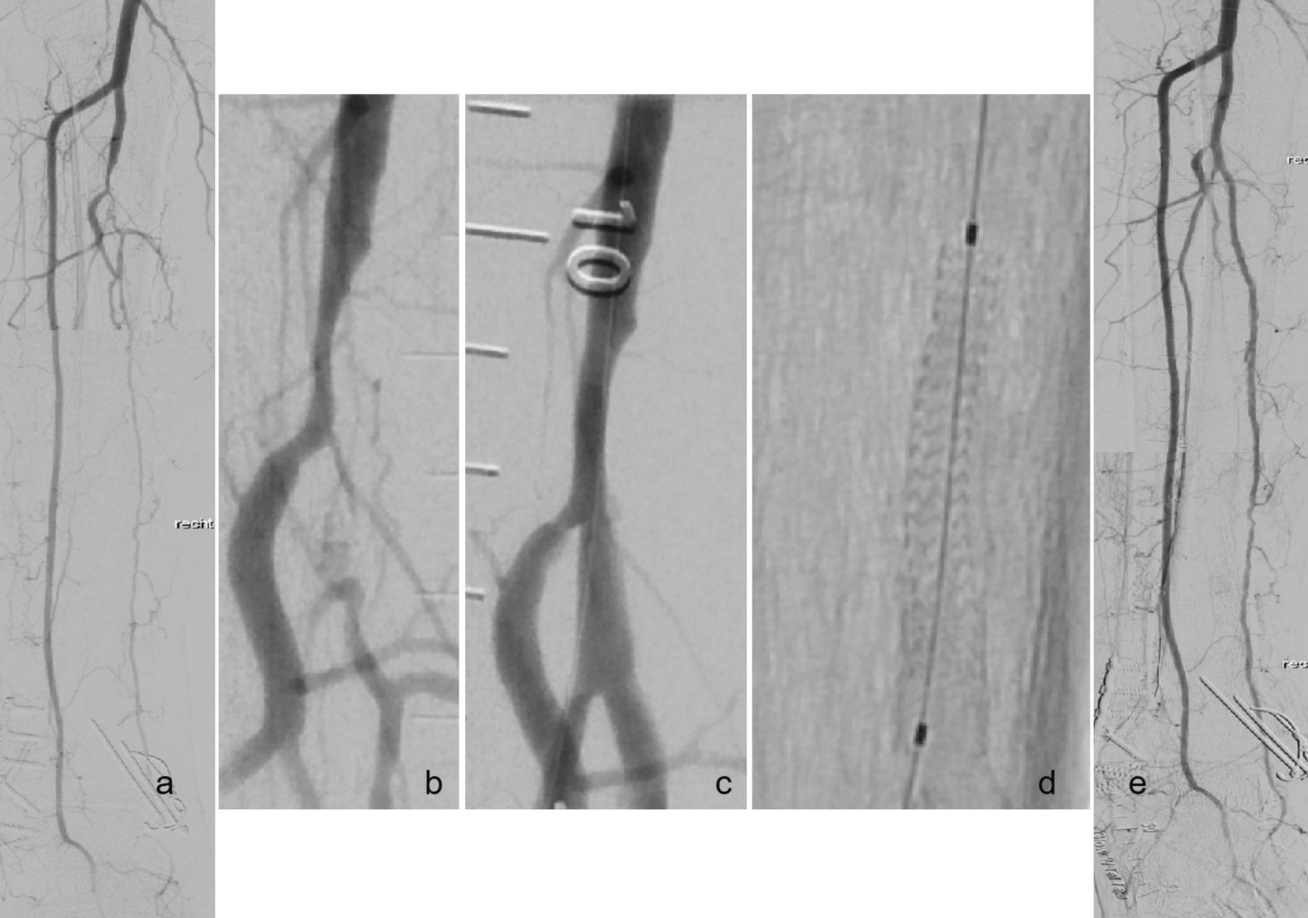
Case example with MOTIV scaffold implantation in tibeoperoneal trunc: **a** pre-intervention, **b** occlusion tibeoperoneal trunc, **c** after pre-dilatation, **d** MOTIV scaffold, **e** final angiography

The evolution of Rutherford class categorization can be seen in Fig. 3. A case example can be seen in Fig. 4.

## Discussion

This non-randomized study demonstrated excellent long-term outcomes, with primary patency rates of 88% at 12 months and 80% at three years. The limb salvage rate of 95% was achieved at three years. Various treatment modalities have been explored for treating BTK disease in patients with CLTI. The results of balloon angioplasty (POBA) have been suboptimal, with primary patency rates of only 50% at one year [2]. Consequently, the use of drug-eluting stents (DES) has been evaluated in numerous trials, proving more effective than POBA for short lesions [8–11]. For example, the DESTINY trial, a randomized controlled trial (RCT) comparing POBA and DES, reported a primary patency rate after 12 months of 85% in the DES group versus 54% in the POBA group for lesions averaging 18.9 ± 10 mm [10]. Similarly, other RCTs, such as YUKON (DES vs. BMS) and ACHILLES (DES vs. POBA), investigated slightly longer lesions (31 ± 9 mm and 27 ± 21 mm, respectively) and demonstrated the superiority of DES in terms of freedom from amputation, target vessel revascularization, and restenosis rates [8, 9]. The DESTINY2 trial extended the analysis to longer lesions, though the mean lesion length remained modest at 47.4 ± 25 mm. It showed a 12-month primary patency rate of 75.4%, a limb salvage rate of 93.6%, and freedom from target lesion revascularization (TLR) of 84.9% [11].

Studies investigating drug-eluting balloons (DEBs) [5–7] have largely failed to demonstrate clinical benefits, likely due to the complexity of BTK disease, which often involves significant elastic recoil, diffuse disease, and frequent calcification.

Drug-eluting bioresorbable scaffolds present an attractive alternative for BTK interventions, combining temporary mechanical support with the release of antiproliferative medication to inhibit neointimal hyperplasia. Three types of bioresorbable scaffolds have been developed:

- *Poly-L-lactic acid (PLLA) scaffolds*: PLLA is a semi-crystalline polymer that degrades into lactic acid upon hydration. While it has been widely used, PLLA scaffolds face limitations, including non-radiopacity, lower tensile and mechanical strength, and the need for thicker, less flexible struts. However, radial strength remains low. [14]
- *Magnesium alloy scaffolds*: Magnesium scaffolds are biocompatible due to the natural presence of magnesium in the body. However, like the PLLA scaffolds, they are not radiopaque, possibly complicating positioning. Early generations of magnesium scaffolds yielded suboptimal results in coronary applications, with higher rates of late lumen loss and ischemia-driven TLR (16.2% vs 5.2%, p = 0.03) [15]. Newer generations, with thinner struts (99–147 μm) and nearly complete (99.6%) degradation within 12 months in a more homogeneous fashion that creates less strut discontinuity, have demonstrated improved outcomes [16].
- *Tyrocore-based scaffolds*: Tyrocore scaffolds feature thin struts (95–115 μm), high radial strength, and radiopacity. Their degradation produces lower levels of lactic acid over a shorter duration, promoting better endothelialization, reduced inflammation, and decreased calcium formation [17].

Varcoe et al. [18] reported promising three-year results for the Absorb scaffold (Abbott Cardiovascular, Inc., Plymouth, MN), an everolimus-eluting bioresorbable PLLA scaffold. In 48 patients with 61 lesions (mean length: 20.1 ± 10.8 mm), the study found an 87.3% freedom from clinically driven TLR (CD-TLR) and a primary patency rate of 81.1% at 36 months. These results are comparable to the current study, which achieved an 80% primary patency rate at three years for slightly longer lesions.

More recently, the LIFE-BTK trial [19] evaluated the Esprit PLLA scaffold versus angioplasty in a multi-center RCT. The study enrolled 261 patients with mean lesion lengths of 43.8 ± 31.8 mm (scaffold group) and 44.8 ± 29.1 mm (angioplasty group). At one year, the primary efficacy outcome measure—a composite of freedom from target limb amputation, target vessel occlusion, CD-TLR, and binary restenosis—was achieved in 74% of the scaffold group versus 44% of the angioplasty group (p < 0.001). However, five scaffold group patients failed the safety outcome measure (freedom from major adverse limb events and perioperative death at six months) compared to none in the angioplasty group. This safety outcome measure was deemed non-inferior compared to angioplasty.

These findings, together with our current study results, suggest that drug-eluting bioresorbable scaffolds outperform the current standard of care (POBA) for BTK lesions. Proper pre-dilation is critical to ensure adequate scaffold expansion and wall apposition.

The main limitations of this study are that it is non-randomized and therefore lacks comparison with other technologies. This reduces the strength of the conclusions. Additionally, the small sample size, exclusion of heavily calcified lesions, and the relatively short lesion length further limit its generalizability. Further research is needed to validate these findings in larger cohorts and longer lesion scenarios.

## Conclusion

The MOTIV sirolimus-eluting Tyrocore-based bioresorbable scaffold showed excellent efficacy and safety results, up to 3 years in CLTI patients with BTK lesions. Patient recruitment has been finished in a larger RCT comparing the MOTIV to angioplasty. These future findings will further help define the role of this technology and will hopefully help improve patient and limboutcomes in CLTI patients.
